# ﻿Morphological and phylogenetic analysis approach to three new species and a new section of *Astragalus* (Fabaceae) from Mongolia

**DOI:** 10.3897/phytokeys.255.140805

**Published:** 2025-04-08

**Authors:** Dariganga Munkhtulga, Shukherdorj Baasanmunkh, Nudkhuu Nyamgerel, Jong Ho Park, Zagarjav Tsegmed, Komiljon Sh. Tojibaev, Hyeok Jae Choi

**Affiliations:** 1 Department of Biology, School of Arts and Sciences, National University of Mongolia, Ulaanbaatar 14201, Mongolia National University of Mongolia Ulaanbaatar Mongolia; 2 Department of Biology and Chemistry, Changwon National University, Changwon 51140, Korea Changwon National University Changwon Republic of Korea; 3 Institute of Botany, Academy of Sciences of Uzbekistan, Tashkent 100125, Uzbekistan Academy of Sciences of Uzbekistan Tashkent Uzbekistan

**Keywords:** *Astragalus*, DNA barcoding, flora of Mongolia, new section, new species

## Abstract

*Astragalus* L. is the largest genus worldwide, comprising more than 3,100 species belonging to 250 sections. In Mongolia, approximately 130 species, including 15 endemic and 25 subendemic species have been previously recognized from 42 sections and 6 subgenera. In this study, we investigated several species within section Laguropsis in Mongolia based on extensive morphological analyses and molecular evidence. Based on these results, we describe three new species and a new section. Two of the newly described species, *A.oyunicus* and *A.teshigicus*, belong to the section Laguropsis, whereas the remaining species, *A.uvsicus*, is the type species of the new section Uvsicus. Furthermore, our findings revealed that (i) *A.tamiricus*, previously considered endemic to Mongolia, is an additional synonym of *A.laguroides*, and (ii) *A.gobi-altaicus*, previously a synonym of *A.laguroides*, is an independent species. Finally, we provide taxonomic nomenclature, morphological observations, distribution maps and wild photo illustrations of each species.

## ﻿Introduction

*Astragalus* L., which belongs to the Fabaceae family, is one of the largest genera of angiosperms, with more than 3,100 species belonging to 250 sections (Podlech and Zarre 2013; POWO 2024). This genus is widely distributed in Asian countries (Maassoumi and Ashouri 2022), particularly in Iran with 850 species followed by 400 in China (Xu and Podlech 2010), 320 in Kazakhstan (Abdulina 1998; Perezhogin et al. 2023), 273 in Uzbekistan (Tojibaev et al. 2015; Sennikov et al. 2016), 270–280 in Kyrgyzstan (Sytin and Lazkov 2018), 150 in Pakistan (Khan et al. 2023), and 90 in Siberia (Vydrina 2006). A recently published checklist of the Tian Shan flora contains at least 222 *Astragalus* species, 82 of which are endemic (Sennikov and Tojibaev 2021). A distinctive feature of the flora of the Mediterranean, Iran, and Central Asia is the rich diversity of species of the genus *Astragalus*. This genus occupies first place in the spectrum of leading genera in the Iran-Turanian flora (Tojibaev et al. 2015). Central Asia is home to at least 650 *Astragalus* species. Morphologically relatively homogeneous and diverging no earlier than the Late Miocene, it comprises some of the highest diversification rates documented thus far in angiosperms (Folk et al. 2024). Taxonomic identification of *Astragalus* is difficult and complicated worldwide. However, a number of new species have been discovered based on morphology (Sytin and Lazkov 2018; Yang et al. 2024) and in combination with morphology and molecular pieces of evidence (Gao et al. 2009; Bagheri et al. 2016, 2022; Erkul et al. 2022). In addition, researchers have studied the seed testa morphology of selected *Astragalus* species to explore their taxonomic significance (Shemetova et al. 2018; Kashyap et al. 2021).

Recently, numerous studies have conducted molecular analyses, such as DNA barcoding, including that on the internal transcribed spacer (ITS), two chloroplast regions (*matK* and *rbcL*) (Gao et al. 2009; Zhang and Jiang 2020; Bagheri et al. 2022, 2023; Erkul et al. 2022; Baasanmunkh et al. 2024a) and comparative complete plastomes (Tian et al. 2021; Moghaddam et al. 2023). According to their research, ITS markers have been successfully identified in most *Astragalus* species, outperforming chloroplast regions (*matK* and *rbcL*) in terms of recognition (Bagheri et al. 2016; Zhang and Jiang 2020; Baasanmunkh et al. 2024a).

In Mongolia, 127 *Astragalus* species belonging to 42 sections and 6 subgenera were recognized (Ulziykhutag 1989, 2003, 2004; Baasanmunkh et al. 2022). Additionally, three new records of *Astragalus* were recently found in the Mongolian flora based on morphological and molecular evidence (Baasanmunkh et al. 2024a, b). In general, *Astragalus* exhibits relatively high endemism in Mongolia compared to other major genera (Baasanmunkh et al. 2021). In particular, 15 and 23 species are currently endemic and subendemic to the country (Baasanmunkh et al. 2021, 2022), respectively.

The section Laguropsis Bunge comprises approximately 44 species distributed in Central Asian countries and Russia (Siberia) (Podlech and Zarre 2013; Tojibaev et al. 2015). In the past, several new species of the section Laguropsis were discovered in Mongolia, including *A.gobi-altaicus* N.Ulziykh (Ulziykhutag 1990) and *A.tamiricus* N.Ulziykh. (in Biazrov et al. 1989). Recently, two new species, *A.admirabilus* Pyak & E.Pyak and *A.liuaiminii* Z.Z.Yang & Q.R.Liu, were described from the Altai Mountains (Russia and Mongolia) (Pyak and Pyak 2019) and Xinjiang (China) (Yang et al. 2024), respectively. In Mongolia, 17 species from the section Laguropsis were reported, including two endemic species (Baasanmunkh et al. 2021; 2022). Among these, *A.gobi-altaicus*, endemic to Mongolia, has been treated as a synonym for *A.laguroides* Pall. by Xu and Podlech (2010).

In the present study, we focused on the *A.laguroides* complex, the most closely related species within the section Laguropsis. Based on our results, we describe three new species and one new section of *Astragalus* from Mongolia based on extensive morphological analysis and nrDNA barcoding.

## ﻿Materials and methods

### ﻿Taxon sampling

We have been conducting field surveys since 2017 to collect fresh samples of *Astragalus*, including detailed wild photographs and leaves across Mongolia. We collected more than 500 herbarium specimens of *Astragalus* that were deposited in the herbaria of the National University of Mongolia (UBU). In addition, we examined herbarium specimens from the following herbaria: ALTB, LE, MW, NS, GFW, TASH, UBA, UBU (Thiers 2023) and FloraGREIF (https://floragreif.uni-greifswald.de). A point distribution map was produced based on the herbarium specimens using ArcGIS (Esri 2012).

### ﻿DNA extraction, amplification, and sequencing

Total genomic DNA was extracted from silica gel-dried leaves using the CTAB method (Doyle and Doyle 1987). A total of 25 samples were extracted from eight species. The nuclear ITS region (White et al. 1990) was used for amplification and sequencing. PCR was performed as previously described (Baasanmunkh et al. 2024a). PCR products were sequenced in both directions by Macrogen (Seoul, Korea). DNA sequences were visually checked and manually trimmed in Geneious Prime 2024.0.7 (www.geneious.com). Automatic alignment of the trimmed sequences using the ClustalW (Thompson et al. 2002) algorithm and construction of a consensus dataset were performed using BioEdit Sequence Alignment Editor v.7.2.5 (Hall et al. 2011). The DNA sequences generated in this study have been deposited in GenBank (www.ncbi.nlm.nih.gov).

### ﻿Phylogenetic analysis

The constructed ITS dataset included 20 *Astragalus* species, including 8 newly sequenced *Astragalus* species from Mongolia, with previously sequenced species from Zhang and Jiang (2020) and Baasanmunkh et al. (2024a). *Phyllolobiumbalfourianum* (N.D.Simpson) M.L.Zhang & Podlech was selected as an outgroup according to Zhang and Jiang (2020). Detailed information on the sample taxa, GenBank accession numbers, and references for each sample are provided in Table 1. Phylogenetic analyses were conducted using maximum parsimony (MP) methods in RAxML v.8.2.11 (Stamatakis 2014) as implemented in Geneious, with the best-scoring maximum likelihood (ML) tree algorithm and 1000 bootstrap replicates. The reconstructed trees were visualized using FigTree v.1.4.2 (Rambaut 2012).

**Table 1.** Detailed information on species, section, subgenus name, GenBank accession numbers, and references of the samples used in this study.

No	Species name	Specimen code	Section	Country	GenBank accession number	Reference
**subgenus Cercidothrix**						
1	*Astragalusdilutus*	UBU0004768	*Laguropsis*	Mongolia	PQ492291	this study
2	*A.dilutus*	UBU0003441	*Laguropsis*	Mongolia	PQ492292	this study
3	*A.dilutus*	UBU0032545	*Laguropsis*	Mongolia	PQ492293	this study
4	*A.dilutus*	UBU0042005	*Laguropsis*	Mongolia	PQ492294	this study
5	*A.dilutus*	UBU0042001	*Laguropsis*	Mongolia	PQ492295	this study
6	*A.dilutus*	UBU0036425	*Laguropsis*	Mongolia	PQ492296	this study
7	*A.dilutus*	UBU0042002	*Laguropsis*	Mongolia	PQ492297	this study
8	*A.teshigicus*	UBU0002138	*Laguropsis*	Mongolia	PQ492298	this study
9	*A.tamiricus*	UBU0039103	*Laguropsis*	Mongolia	PQ492299	this study
10	*A.tamiricus*	UBU0010820	*Laguropsis*	Mongolia	PQ492300	this study
11	*A.tamiricus*	UBU0040555	*Laguropsis*	Mongolia	PQ492301	this study
12	*A.laguroides*	UBU0026340	*Laguropsis*	Mongolia	PQ492302	this study
13	*A.laguroides*	UBU0026328	*Laguropsis*	Mongolia	PQ492303	this study
14	*A.laguroides*	UBU0006948	*Laguropsis*	Mongolia	PQ492304	this study
15	*A.laguroides*	UBU0039105	*Laguropsis*	Mongolia	PQ492305	this study
16	*A.gobi-altaicus*	UBU0012226	*Laguropsis*	Mongolia	PQ492314	this study
17	*A.gobi-altaicus*	UBU0030804	*Laguropsis*	Mongolia	PQ492315	this study
18	*A.ochrias*	UBU0006593	*Laguropsis*	Mongolia	PQ492306	this study
19	*A.lupulinus*	UBU0026235	*Laguropsis*	Mongolia	PQ492310	this study
20	*A.oyunicus*	UBU0034202	*Laguropsis*	Mongolia	PQ492311	this study
21	*A.oyunicus*	UBU0039102	*Laguropsis*	Mongolia	PQ492312	this study
22	*A.oyunicus*	UBU0014416	*Laguropsis*	Mongolia	PQ492313	this study
23	*A.uvsicus*	UBU0039100	*Uvsicus*	Mongolia	PQ492307	this study
24	*A.uvsicus*	UBU0039101	*Uvsicus*	Mongolia	PQ492308	this study
25	*A.uvsicus*	UBU0027685	*Uvsicus*	Mongolia	PQ492309	this study
26	*A.ammodytes*		*Ammodytes*	Mongolia	OR527932	Baasanmunkh et al. (2024a)
27	*A.gubanovii*		*Macrotrichoides*	Mongolia	OR527933	Baasanmunkh et al. (2024a)
28	*A.testiculatus*		*Mixiotricha*	Mongolia	OR527928	Baasanmunkh et al. (2024a)
29	*A.hypogaeus*		*Trachycercis*	Mongolia	OR527930	Baasanmunkh et al. (2024a)
30	*A.junatovii*		*Trachycercis*	Mongolia	OR527929	Baasanmunkh et al. (2024a)
31	*A.teskhemicus*		*Trachycercis*	Mongolia	OR527931	Baasanmunkh et al. (2024a)
**subgenus Hypoglottis**						
32	*A.danicus*		*Hypoglottis*	Russia	OQ106945	Unpublished
33	*A.laxmannii*		*Hypoglottis*	China	MT923540	Unpublished
34	*A.tibetanus*		*Hypoglottis*	Kazakhstan	OQ106946	Unpublished
s**ubgenus *Astragalus***						
35	*A.bhotanensis*		*Brachycephali*	China	MF044289	Zhang and Jiang (2020)
36	*A.skythropos*		*Skythropos*	China	MF044266	Zhang and Jiang (2020)
37	*Phyllolobiumbalfourianum*			China	MF044295	Zhang and Jiang (2020)

## ﻿Results and discussion

We partially revised the section Laguropsis belonging to the subgenus Cercidothrix in Mongolia based on morphological and molecular analyses in the present study. Here, we described three new species, *A.oyunicus*, *A.teshigicus*, and *A.uvsicus*, from eastern, northern, and western Mongolia. In addition, we described a new section, *Uvsicus*, based on *A.uvsicus*. Furthermore, the taxonomic status of some species, such as *A.tamiricus*, was previously endemic to Mongolia and an additional synonym of *A.laguroides*. In contrast, *A.gobi-altaicus* was synonymous with *A.laguroides*, an independent species from *A.laguroides*.

### ﻿DNA barcoding

**Figure 1.**
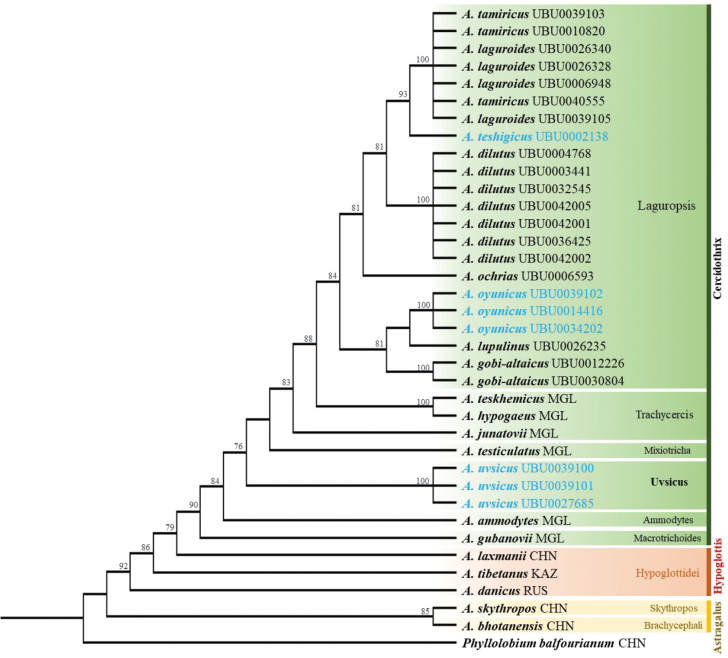
Phylogenetic tree of *Astragalus* species based on ITS sequences. Newly sequenced samples are indicated with UBU specimen code. The bootstrap support values above 70% are shown at branch level. CHN, MGL, KAZ, and RUS samples are from China, Mongolia, Kazakhstan, and Russia, respectively.

The aligned ITS region was 598 bp long with 174 variable characteristics, 21 of which were parsimony informative. The ITS1 sub-region included an 11 bp parsimony-informative site, which was slightly variable compared to the others. The phylogenetic tree showed that the ITS sequence data supported the monophyletic status of the genus *Astragalus* with strong bootstrap support (Fig. 1). A newly sequenced *Astragalus* species from Mongolia nested within a species from the subgenus Cercidothrix. Molecular phylogenetic studies show that most *Astragalus* is monophyletic (Kang et al. 2003). Our phylogenetic results showed the same topology as in a previous study.

### ﻿Taxonomic treatment

**Figure 2.**
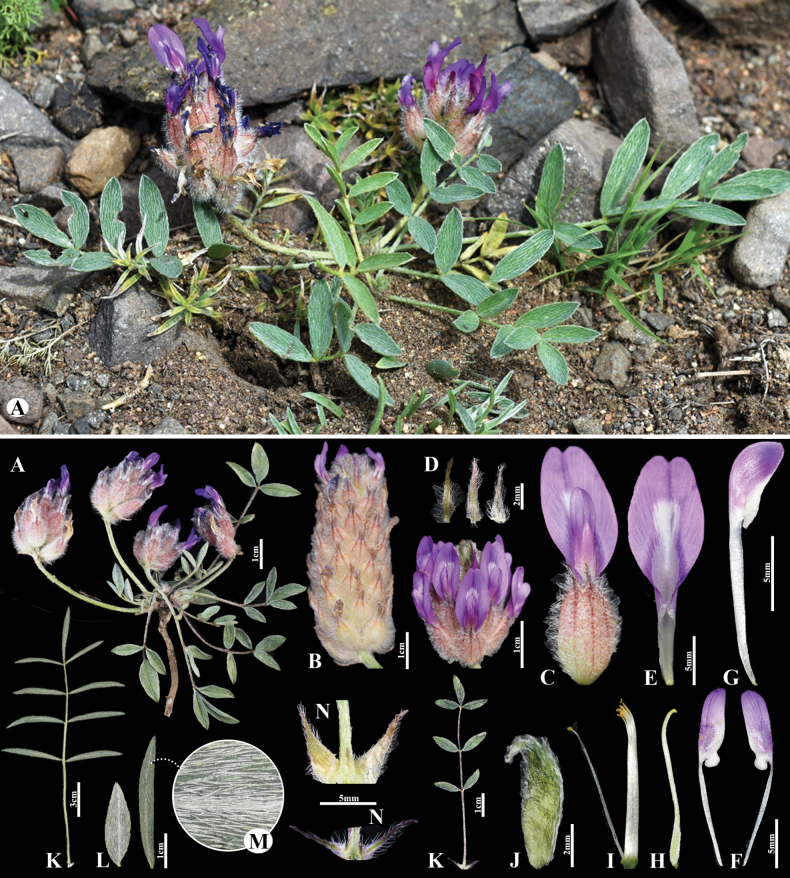
*Astragaluslaguroides* in Mongolia **A** general habit **B** raceme **C** flower **D** bracts **E** standard **F** wings **G** keel **H** pistil **I** stamens **J** pod **K** leaves **L** leaflet **M** omission of medifixed hairs on the upper side of the leaf **N** stipules. (Photo credits: S.Baasanmunkh and D.Munkhtulga).

**AstragalussubgenusCercidothrixBunge,sectionLaguropsis Bunge, Mém. Acad. Imp. Sci. Saint Pétersbourg 11(16): 137 (1868). (Fig. 2)**

#### ﻿ Astragalus laguroides

Taxon classificationPlantaeFabalesFabaceae

Pall., Reise Russ. Reich. 3(2): App. 750 (1776).

833B6C05-5519-501B-8B4C-150A39B40FCB

Fig. 2

≡ Astragaluslagurus Pall., Sp. Astragal.: 18 (1800).
≡ Saccocalyxlaguroides (Pall.) Steven, Bull. Soc. Imp. Naturalistes Moscou 4: 269 (1832).
≡ Tragacanthalaguroides (Pall.) Kuntze, Revis. Gen. Pl. 2: 945 (1891).
= Astragalustamiricus N.Ulziykh. in L.G.Byazrov & al., Fl. Khangaya: 124 (1989). syn. nov. • Type. Mongolia. Khangai region: • Arkhangai province, Ikhtamir soum, Khoid Tamir river, monasterium Tzetza-Van, shrublands, 19 August 1926, *N. Pavlov* (Holotype LE01017820!, Paratype LE01017821!, Paratype LE01017822!, Isotype MW0593230!).

##### Type.

Mongolia. circa Selenga river, *P.S. Pallas* [BM! (Fig. 6D)].

##### Diagnosis.

*Astragaluslaguroides* is a type species in the section Laguropsis (Podlech and Zarre 2013). This species is widely distributed in central and western Mongolia. According to the description of *A.tamiricus*, some morphological characteristics, such as racemes, leaflets, stipules, and flowers, including calyx, standard, keel, and wings, are slightly smaller than *A.laguroides* (Biazrov et al. 1989). However, our studied samples of *A.tamiricus*, collected from the type location in Arkhangai Province, clustered with *A.laguroides* based on our phylogenetic tree (Fig. 1). Therefore, we treated *A.tamiricus* as an additional synonym of *A.laguroides* in this study.

##### Description.

Plants perennial 8–15(–20) cm tall, acaulescent or nearly so, in vegetative parts covered with medifixed, appressed white hairs. Stems, if present, up to 1 cm, angular-sulcate, densely hairy. Stipules 6–8(–10) mm, narrowly triangular-acuminate, adnate to the petiole for c. 3–4 mm, otherwise free from each other, covered with spreading or ascending hairs. Leaves 3–14(–18) cm; petiole 1.5–5(–8) cm, like the rachis rather densely to densely, more rarely loosely hairy. Leaflets in 3–5(–8) pairs, in the basal leaves often only in 1–2 pairs, narrowly elliptic to more rarely elliptic, 8–25(–40) × 3–6 mm, acute, on both sides rather densely appressed hairy. Peduncles 2–12 cm, rather densely covered with symmetrically to asymmetrically bifurcate, appressed to subappressed white hairs, toward the raceme sometimes also with some black hairs mixed in. Racemes ovoid, 2–4(–6) cm long, densely many-flowered. Bracts linear-acute, 4–8 mm, covered with asymmetrically bifurcate, at the margins with basifixed white hairs. Calyx at anthesis tubular, soon becoming ovoid-inflated, 11–14 mm, loosely to rather densely covered with asymmetrically bifurcate to basifixed, tangled, spreading white hairs 1–2 mm, at outer side of the teeth and sometimes at the nerves of the tube also with black hairs; teeth subulate, 3–4 mm. Petals violet. Standard 17–20 mm; blade 6–7 mm wide, narrowly obovate, apex emarginate. Wings slightly shorter than standard, ca. 14–18 mm long; blades narrowly oblong, rounded, 5.5–7 × 1.5–2 mm; auricle 1 mm, claw 9–10 mm. Keel 13–15 mm; blades 4–4.5 × 1.8–2.2 mm; claw 9–10 mm. Ovary sessile, narrowly ellipsoid. Pods enclosed in the calyx, sessile, oblong, (6) 7−8 × 2−3 mm long, keeled ventrally, slightly and widely grooved dorsally, with a hooked beak c. 1 mm, unilocular; covered with subappressed to spreading white hairs 1–1.5 mm, smaller black hairs amount to greater than the apex.

##### Distribution.

China (Inner Mongolia, Xinjiang), Russia and Mongolia (Podlech and Zarre 2013) (Fig. 9).

##### Habitat.

This species grows in debris, stony slopes, trails, pebble beds, rocky and stony screes.

##### Additional specimens examined.

Mongolia. Khuvsgul region: • Selenge province, 13 km west from Tsagaannuur soum, 50°6'0"N, 105°21'0"E, 757 m, 12 June 2012, *D. N. Shaulo* (NS0010107) • Khangai region: Khangai, at the spring between Oit-beise and Lugan-kure, 47°48'13.17"N, 101°58'23.88"E, 1471 m, 11 July 1926, *N. Ikkonnikov-Galitzky* (LE01017821) • Arkhangai province, Tuvshruulekh soum, 47°26'43.6"N, 101°55'9.5"E, 1572 m, 20 June 1977, *N. P. Guricheva* (MW0183266, MW0183267) • Arkhangai province, Tsenkher soum, Urd Tamir river, 48°23'59.9"N, 97°9'4.9"E, 2118 m, 11 July 1978, *I. A. Gubanov* (MW0183264) • Bulgan province, Khishig-Undur soum, Teg river, 48°48'17.9"N, 103°31'49.4"E, 1209 m, 05 July 1974, *O. V. Jurba* (MW0183270) • Bulgan province, Mogod soum, Nomgon mountain, 48°16'1.17"N, 102°55'44.67"E, 1706 m, 02 July 1980, *Sh. Dariimaa* (UBA) • Bulgan province, 9 km south east from Unit soum, 49°15'15"N, 102°56'41.6"E, 1238 m, 17 June 1964, *Khishgee* (UBA) • Bulgan province, Khangal soum, Burgastain davaa, 49°10'22.99"N, 104°28'38.27"E, 1433 m, 04 August 1978, *B. Mandakh & Sh. Dariimaa* (UBA). Arkhangai province, Khangai soum, 47°50'0.132"N, 99°21'6.876"E, 2135 m, 05 August 2020, *Sh. Baasanmunkh* (UBU0010817, UBU0032212) • Arkhangai province, Battsengel soum, 47°45'53.208"N, 101°56'38.904"E, 1420 m, 20 August 2020, *Sh. Baasanmunkh* (UBU0010820) • Arkhangai province, Ikh Tamir soum, Tamir river, 47°36'27.9"N, 101°8'17.3"E, 1420 m, 30 June 2023, *D. Munkhtulga* (UBU0039991–UBU0039998, UBU0040555, UBU0040556) • Arkhangai province, Ikh Tamir soum, Tamir river, 1666 m, 28 May 2024, *B. Oyuntsetseg, G. Bayarmaa & D. Munkhtulga* (UBU0039103, UBU0039104) • Dauria region: • Tuv province, 3 km northwest from Bornuur soum, 48°32'7.3"N, 106°8'1.5"E, 1208 m, 07 June 1973, *K. Kloss* (GFW45636) • Tuv province, Unjuul soum, Argal mountain, 47°59'8.22"N, 105°55'2.1"E, 1445 m, 19 July 1973, *Ch. Sanchir* (UBA) • Selenge province, Dulaankhan soum, Yruu river, Dulaankhan mountain, 49°56'3.1"N, 106°12'21.5"E, 750 m, 09 August 1982, *E. Ganbold* (UBA) • Mongolian Altai region: • Khovd province, Darvi soum, Sutai mountain, 46°23'59.1"N, 94°0'38.3"E, 1900 m, 18 August 1984, *I. A. Gubanov* (MW0183284) • Gobi-Altai province, Tsetseg soum, 46°19'3.817"N, 93°2'35.239"E, 1430 m, 12 July 2019, *B. Oyuntsetseg* (UBU0006948) • Gobi-Altai province, Tseel soum, Baga Tayan, 45°24'54"N, 95°52'4.8"E, 1893 m, 07 July 2019, *B.Oyuntsetseg*, *Sh.Baasanmunkh* (UBU0005920, UBU0005922) • Middle Khalkh region: • Avzaga mountain, 48°1'53.05"N, 103°14'40.64"E, 1917 m, 12 June 1971, *V. I. Grubov, N. Ulziikhutag & Sh. Dariimaa* (UBA) • Tuv province, 7 km south from Unjuul soum, 46°49'12"N, 105°30'36"E, 1552 m, 30 July 1973, *T. I. Kazantseva* (NS0025349) • same locality, 7 June 1974, *T. K. Gordeeva*, (NS0025347, NS0025348) • Tuv province, Buren soum, Ikher-Uhaa, 46°59'13.9"N, 105°3'28.1"E, 1377 m, 22 July 1976, *Tumurtogoo* (UBU0039987) • Depression of Great Lakes region: • Khovd province, Myangad soum, 48°15'7.898"N, 91°54'14"E, 1185 m, 26 August 1984, *I. A. Gubanov* (MW0183279) • Khovd province, Chandmani soum, Jargalant khairkhan mountain, 47°49'23.2"N, 92°24'15.1"E, 1836 m, 19 August 2003, *M. Schnittler* (GFW45638) • Khovd province, 5 km south west from Khar-Us Lake, 47°42'59.8"N, 91°58'59.902"E, 1459 m, *S. A. Dyachenko & P. A. Kosachev* (ALTB1100016497) • Valley of Lakes region: Bayankhongor province, 30 km west from Arvaikheer city, 46°3'50.4"N, 102°30'18"E, 2130 m, 1974, *Ts. Jamsran*, (UBU0026328) • Uvurkhangai province, Guchin-Us soum, 45°24'8.399"N, 102°14'55.298"E, 1575 m, 23 July 1978, *G. N. Ogureeva* (MW0183268) • Bayankhongor province, Bogd soum, 45°31'30"N, 101°6'28.8"E, 1569 m, 12 July 2019, *Batsaikhan* (UBU0006948) • Bayankhongor province, Bumbugur soum, 46°13'8.4"N, 99°6'43.2"E, 1994 m, 05 July 2019, *B. Oyuntsetseg, Sh. Baasanmunkh* (UBU0006680) • Bayankhongor province, Buutsagaan soum, 46°11'36.5"N, 99°10'1.5"E, 1880 m, 29 May 2024, *B. Oyuntsetseg, G. Bayarmaa & D. Munkhtulga* (UBU0039105–UBU0039112) • Gobi Altai region: • Bayankhongor province, Buutsagaan soum, south slope of Bogd mountain, 44°57'42.38"N, 100°12'10.31"E, 2725 m, 02 July 1977, *E. Ganbold* (UBA) • Bayankhongor province, Bogd soum, Orog lake, 44°59'19.1"N, 100°52'50.999"E, 1100 m, 16 July 2013, *R. Tungalag & Ts. Tsendeekhuu* (UBU0021615) • Trans-Altai Gobi region: • Bayankhongor province, Shinejinst soum, Djinst mountain, Altai tsagaan khaalga, 44°25'37.582"N, 99°19'6.24"E, 2190 m, 07 September 1979, *Ch. Sanchir* (UBA) • Govi-Altai province Tseel soum, Aj Bogd mountain, Ar zuslan river, 44°41'12.221"N, 95°14'9.481"E, 2400 m, 09 August 1984, *Kh. Buyan-Orshikh & Yu. G. Evtipeev* (UBA) • Bayankhongor province, Shinejinst soum, Djinst mountain, 44°26'28.4"N; 99°13'36"E, 2000 m, 14 July 1979, *I. A. Gubanov* (MW0593124, MW0593125).

#### ﻿ Astragalus gobi-altaicus

Taxon classificationPlantaeFabalesFabaceae

N.Ulzyikh., Byull. Moskovsk. Obshch. Isp. Prir., Otd. Biol., n.s., 95(2): 83 (1990).

F53FE1E1-AEB1-586E-8FBF-B0018BD2450F

Fig. 3

##### Diagnosis.

*Astragalusgobi-altaicus* was first described in East Gobi, Mongolia (Ulziykhutag 1990). Later, this species was treated as a synonym of *A.laguroides* by Xu and Podlech (2010). However, based on our extensive morphological studies, *A.gobi-altaicus* can be distinguished from *A.laguroides* by its inflorescence being oblong-cylindrical (vs. globose or ovate), leaflets that are broadly elliptical or oblong-obovate, more rarely sub-oval (vs. leaflets oblong, narrowly elliptical, or lanceolate), calyx teeth 4–6 mm long (vs. calyx teeth 3–4) (Table 2). In addition, the phylogenetic tree supported that *A.gobi-altaicus* was different from *A.laguroides*, which was more similar to *A.oyunicus* and *A.lupulinus* (Fig. 1).

**Table 2.** Morphological comparisons of five *Astragalus* species in Mongolia.

Characters	*A.laguroides*	*A.gobi-altaicus*	*A.oyunicus*	*A.teshigicus*	*A.uvsicus*
Leaves	3−14(−18) cm long	8−14 cm long	5−12(−15) cm long	8−18 cm long	3−8(−12) cm long
Leaflets	3−5(−8) pairs, narrowly elliptic, 8−25(−40) × 3−6 mm, both surfaces rather densely hairy, apex acute	2−4 pairs, broadly-elliptical or oblong-obovate, 7−12 × 3–5 mm, on both sides densely hairy, apex mucronate	3−5 pairs, narrowly elliptic, 15−18 × 4−7 mm, on both sides densely hairy, apex acute	4−6 pairs, elliptical, 14−20 × 5−8 mm, on both sides densely covered with medifixed, subappressed hairs, acute to rarely obtuse	2−5 pairs, narrowly elliptic, 8−11(−14) × 3−5 mm, rather densely covered with medifixed, appressed hairs, apex acute
Stipules	5−8(−10) mm long, narrowly triangular-acuminate, covered with spreading or ascending hairs	3−4 mm long, narrowly triangular-acuminate, rather densely appressed hairy, at the margins with basifixed hairs	4−6 mm long, broadly triangular-acuminate, loosely covered with spreading or ascending hairs	4−5 mm long, broadly triangular-acuminate, densely covered with spreading or ascending hairs	6−8 mm long, narrowly triangular-acuminate, covered with spreading or ascending hairs
Raceme	ovoid to oblong, 2−4(−6) cm long	oblong-cylindrical, 3−8 cm long	ovoid to oblong, 3−4 cm long	oblong or oblong-cylindrical, 4−7(−8) cm long	globose or ovate, 3−4 cm long
Calyx	11−14 mm long, densely covered with asymmetrically bifurcate to basifixed, tangled, spreading white hairs 1–2 mm, at outer side of the teeth and sometimes at the nerves of the tube also with black hairs; teeth 3−4 mm	12−16 mm long, densely covered with subbasifixed to basifixed, spreading, straight white hairs 2−3 mm; teeth 4−6 mm	10−12 mm long, covered with basifixed, spreading, straight white and fewer black hairs 1−2 mm; teeth 2−3 mm	12−15 mm long, rather densely covered with spreading, straight white hairs 2 mm; teeth 2−3 mm	11−14 mm long, covered with basifixed, spreading white and black hairs 1−2 mm; teeth 2−3 mm
Standard	17−20 mm long, emarginate	14−18(−20) mm long, slightly emarginate	18−20 mm long, emarginate	20−24 mm long, emarginate	15−18 mm long, emarginate
Wings	14−18 mm long	12−16 mm long	14−18 mm long	17−22 mm long	13−15 mm long, obtuse
Keels	13−15 mm long	10−14 mm long	12−16 mm long	14−18 mm long	12−14 mm long
Pods	sessile, oblong, (−6)7−8 × 2−3 mm, with a hooked beak c. 1 mm, unilocular; covered with subappressed to spreading white hairs 1−1.5 mm, smaller black hairs amount greater than the apex	sessile, oblong, 7−8 × 3−4 mm, with a hooked beak 2−2.5 mm, unilocular; covered with tangled, spreading white hairs	sessile, oblong, 6−7 × 3−4 mm, with a hooked beak c. 1 mm, unilocular; densely covered with spreading white and fewer black hairs c. 2 mm	sessile, oblong, 5−6 × 2−3 mm, with a hooked beak c. 1 mm, unilocular; densely covered with spreading white hairs	sessile, linear, 6.5−7 × 2−3 mm, with beak c. 1 mm, bilocular, loosely covered with spreading white hairs c. 1 mm, smaller black hairs amount greater than the apex

##### Type.

Mongolia. Umnugovi province, Noyon soum, Noyon-Bogd mountain, 08 September 1979, *V. I. Grubov, A. Muldaschev & Sh. Darijmaa 1964* [Holotype LE01016096! (Fig. 6F)] • Umnugovi province, Nomgon soum, Khurkh mountain, 42°43'16.6"N, 105°06'17.4"E, 1425 m, 28 June 1980, *I. A. Gubanov 5941* [Paratype MW0593122!] • Umnugovi province, Khurmen soum, Bayan-Undur, 42°33'18.1"N, 103°54'38.1"E, 1413 m, 06 August 1981, *I. A. Gubanov 3376* [Paratype MW0593121!, MW0593120!].

##### Description.

Plants perennial 8–12 cm tall, acaulescent, with merely white, in vegetative parts distinctly warty hairs. Stipules whitish, 3−4 mm, triangular, nearly free from the petiole, not connate behind the stem, appressed hairy, at the margins with basifixed hairs. Leaves (–3)8–14 cm; petiole 1–4 cm, like the rachis slender, loosely to rather densely covered with medifixed, appressed hairs 0.5−1 mm. Leaflets in 2−4 pairs, in the basal leaves often in 1–2 pairs only, oblanceolate or narrowly elliptic to elliptic, 7−18 × 5–10 mm, mucronate, on both sides densely covered with medifixed, appressed hairs 1.5−2(−3) mm. Peduncles 3−6 cm, slightly angular-sulcate, loosely to rather densely covered with medifixed, appressed hairs, partly glabrescent with age. Raceme oblong to oblong-cylindrical 3−8 cm long, densely many-flowered. Bracts whitish, 3−5 mm, narrowly linear, covered with mostly basifixed hairs. Calyx 11−14 mm, tubular at beginning of anthesis, soon ovoid-inflated, rather densely covered with subbasifixed to basifixed, rigid, spreading, straight white hairs 2−3 mm; teeth filiform, 4−5 mm. Standard (–9)14−18(–20) mm; blade c. 5 mm wide, elliptic, narrowly triangular toward the slightly emarginate tip, slightly constricted below the middle, at the base obtusely angularly passing into the claw. Wings 12–15 mm; blades narrowly oblong, obtuse, c. 5.5 × 1.5 mm; auricle 1 mm, claw 8 mm. Keel 10–12 mm; blades obliquely obovate 3.5 × 2 mm; claw c. 8 mm. Ovary sessile. Pods enclosed in the calyx, sessile, oblong, 7−8 × 3−4 mm long, with a hooked beak c. 2−2.5 mm, unilocular; covered with tangled, spreading only white hairs.

**Figure 3.**
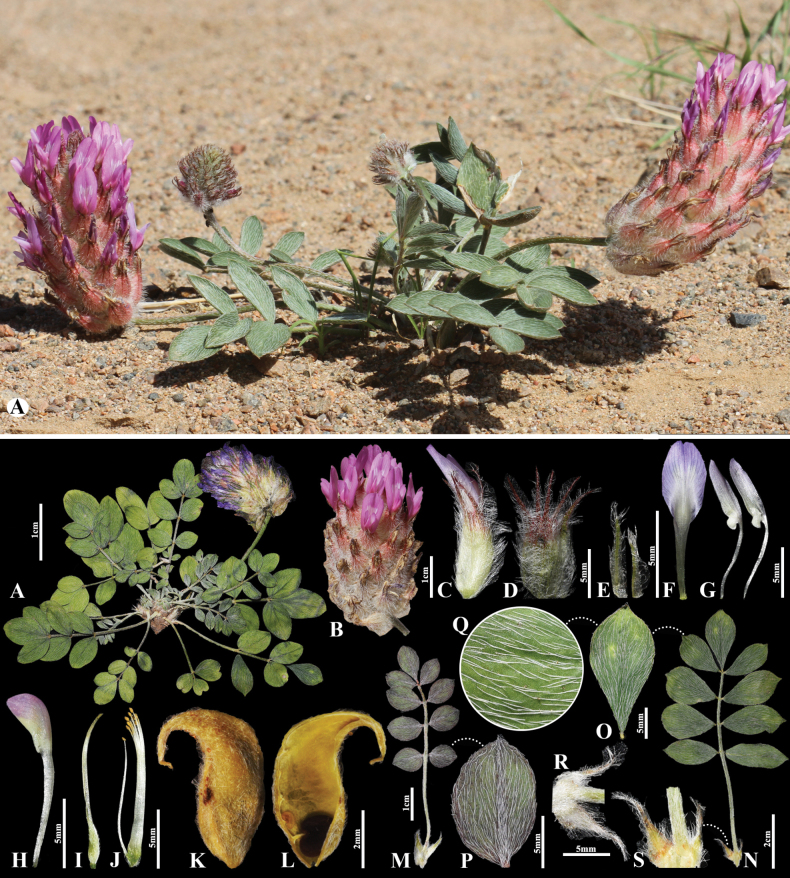
*Astragalusgobi-altaicus* in Mongolia **A** general habit **B** raceme **C** flower **D** calyx **E** bracts **F** standard **G** wings **H** keel **I** pistil **J** stamens **K** pod **L** pod valve **M** leave, abaxial view **N** leave, adaxial view **P** leaflet, abaxial view **O** leaflet, adaxial view **Q** omission of medifixed hairs on the upper side of the leaf **R** stipules, outside view **S** stipules, inside view. (Photo credits: D.Munkhulga and B.Oyuntsetseg).

##### Distribution.

Endemic to Mongolia. This species is currently known from the South Gobi in Mongolia (Fig. 9).

##### Habitat.

This species grows in debris desert steppe, mountain slopes, sandy desert steppes, sayr slopes and granite slopes of hillocky areas.

##### Additional specimens examined.

Mongolia, Gobi Altai region: • Umnugovi province, Tsogt-Tsetsii soum, Tsetsii mountain, 43°30'48.38"N, 105°41'18.85"E, 1727 m, 28 May 1962 (UBA) • Umnugovi province, Nomgon soum, Baruun tsohiotiin Khudag, 42°41'14.1"N, 105°13'8.5"E, 1463 m, 17 May 1962 (UBA) • Umnugovi province, Bulgan soum, Dundsaikhan mountain, Khaalgiin sair, 43°44'16.53"N, 103°34'10.88"E, 2229 m, 25 June 1962 (UBA) • Umnugovi province, 20 km southeast from Khurmen soum, 43°5'55.7"N, 104°11'39.4"E, 1631 m, 06 July 1973, *Ts. Jamsran* (UBU0026387) • Umnugovi province, Bayandalai soum, northern part of Zuramtai mountain, 1715 m, 05 September 1976, *Ch. Sanchir* (UBA) • Bayankhongor province, Shinejinst soum, Jinst mountain, 44°26'28.4"N, 99°13'36"E, 2000 m, 14 July 1979, I. *A. Gubanov* (MW0593124, MW0593125) • Umnugovi province, Noyon soum, Noyon Bogd mountain, 43°12'17.35"N, 101°50'39.68"E, 2200 m, 08 September 1979, *V. I. Grubov & Sh. Dariimaa* (UBA) • Umnugovi province, Sewrei soum, Zuulun mountain, 43°38'49.19"N, 102°20'57.46"E, 1900 m, 26 August 1982, *I. A. Gubanov* (MW0593123) • Umnugovi province, Dalanzadgad soum, Khachig mountain, 42°59'35.53"N, 105°40'35.27"E, 1800 m, 01 September 1982, *I. A. Gubanov* (MW0183265) • Umnugovi province, Khankhongor soum, Chanaijn Kharaa mountain, 44°22'48"N, 104°16'48"E, 1242 m, 22 June 1988, *H. D. Knapp* (GFW45639) • Umnugovi province, Bayandalai soum, Ikh Argalant mountain, 43°10'32.24"N, 103°35'24.93"E, 1910 m, 16 June 2007, *Ts. Jamsran* (UBU0030804) • Umnugovi province, Bayan-Ovoo soum, 42°56'57.5"N, 106°9'13.3"E, 1170 m, 08 June 2013, *Ch. Sanchir* (UBU0032442) • Alashan Gobi region: • Umnugovi province, Alashaa Gobi, Khalzan mountain, 42°10'18.32"N, 105°15'43.97"E, 1363 m, 31 July 1989, *I. A. Gubanov* (MW0183261) • Umnugovi province, Nomgon soum, Borzon govi, 42°33'35.14"N, 105°9'58.8"E, 1208 m, 2003, *Ariuntuya* (UBU0012226).

#### ﻿ Astragalus oyunicus

Taxon classificationPlantaeFabalesFabaceae

D.Munkhtulga & S.Baasanmunkh sp. nov.

BE814C3F-EABD-52A7-AAEE-A539783EF3A3

urn:lsid:ipni.org:names:77359973-1

Fig. 4

##### Diagnosis.

The new species is close to *A.gobi-altaicus*, but differs by its leaves having leaflet narrowly elliptic, 15–18 × 4–7 mm, apex acute (vs. oblanceolate or narrowly elliptic to elliptic, 7–18 × 5–10 mm, apex mucronate) and raceme oblong to oblong-cylindrical, 3–8 cm long (vs. ovoid to oblong, 3–4 cm long). It is also similar to *A.laguroides* in leaf shape and general habit, but differs in its stipules broadly triangular-acuminate, 4–6 mm long (vs. narrowly triangular-acuminate, 5–8(–10) mm long) and pod densely covered with spreading white and fewer black hairs 2 mm (vs. covered with subappressed to spreading white hairs 1–1.5 mm, smaller black hairs amount greater than the apex) (Table 2).

**Figure 4.**
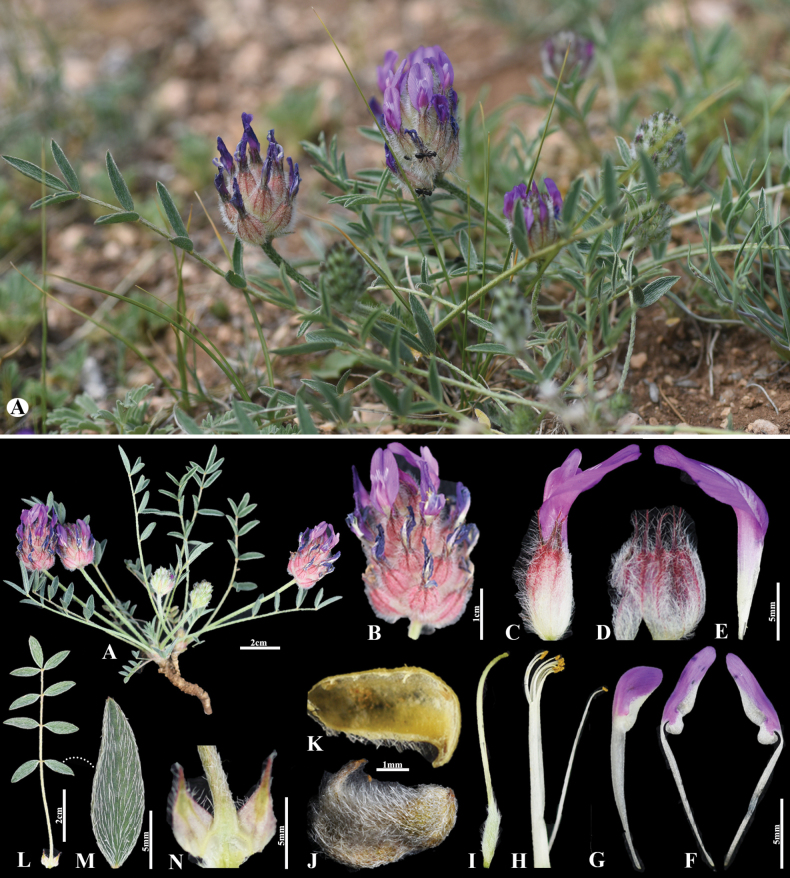
*Astragalusoyunicus* in Mongolia **A** general habit **B** raceme **C** flower **D** calyx **E** standard **F** wings **G** keel **H** stamens **I** pistil **J** pod **K** pod valve **L** laeve **M** leaflet **N** stipules. (Photo credits: D.Munkhtulga).

##### Type.

Mongolia. Khentii province, Bayankhutag soum, 46°54'10.2"N, 110°12'51.9"E, 1166 m, 05 August 2023, *B. Oyuntsetseg & D. Munkhtulga Khen-2023* [Holotype UBU0039102! (Fig. 6C)] • Ulaanbaatar city, Bayanzurkh district, Gachuurt village, 47°55'52.3"N, 107°07'51.2"E, 1467 m, *Yondon* [Isotype UBU0034202!].

##### Description.

Plants perennial, 5−15 cm tall, acaulescens, in vegetative parts covered with ± medifixed, appressed white hairs c. 1 mm. Rootstock divided with short to elongated blackish brown branches. Stipules broadly triangular-acuminate, 4−6 mm, shortly adnate to the petiole, otherwise free from each other, densely covered with hairs up to 1.5 mm, at the margins also with basifixed hairs. Leaves 5−12(−15) cm; petiole 2−4 cm, like the rachis covered with medifixed, appressed hairs. Leaflets in 3−5 pairs, narrowly elliptic, 15−18 × 4−7 mm, on both sides densely hairy, apex acute. Peduncles 3−7 cm, rather densely to densely covered with symmetrically to asymmetrically bifurcate, flexuose, subappressed white hairs. Racemes ovoid to oblong 3−4 cm long. Bracts whitish, 5−6 mm, narrowly triangular, with strongly asymmetrically bifurcate, ± spreading white hairs. Calyx at beginning of anthesis tubular, later on ovoid-inflated, 10−12 mm long, covered with basifixed, spreading, straight white and fewer black hairs 1−2 mm; teeth subulate, 2−3 mm. Petals violet. Standard 18−20 mm; blade 5−6 mm wide, obovate, slightly constricted in the middle, emarginate, at the base gradually narrowed. Wings 14−16 mm; blades narrowly oblong, obtuse, 5.5−6.5 × 2−2.5 mm; auricle c. 1.5 mm; claw c. 9 mm. Keel 12−14 mm; blades 4−4.5 × 3 mm; claw 8−8.5 mm. Ovary sessile. Legumes enclosed in the calyx, 6−7 × 3−4 mm, with a hooked beak c. 1 mm, unilocular; densely covered with spreading white and fewer black hairs c. 2 mm.

##### Distribution.

Endemic to Mongolia (Fig. 9).

##### Habitat.

This species grows in stony mountain slopes and low isolated rounded hills of the steppe region.

##### Etymology.

The species is named after Prof. Batlai Oyuntsetseg who is a botanist in Mongolia.

##### Additional specimens examined.

Mongolia. Mongolian Dauria region: • Ulaanbaatar city, Chingeltei district, Zuun salaa, 23 August 1963 (UBU0032447) • Ulaanbaatar city, Narangiin enger, 25 July 1967, *L. Purevsuren* (UBU0026331, UBU0026338) • Ulaanbaatar city, Songinokhairkhan district, Tolgoit, Narangiin enger, 10 August 1967, *Tsogoo* (UBU0026224, UBU0032448, UBU0032449, UBU0039988-UBU0039990) • Khentii province, Jargalant Khan soum, Kherlen river, 07 July 1979, *V.I. Grubov & A. Mundaashev 321* (UBA) • Ulaanbaatar city, Chingeltei district, Shadivlan, 48°01'00"N, 106°54'01"E, 1520 m, 06 June 2021, *B. Oyuntsetseg & A. Anudari* (UBU0014416) • Middle Khalkh region: Tuv province, Buren soum, Ikher-Ukhaa, 22 July 1976, *Tumurtogoo* (UBU0039987) • Govisumber province, Sumber soum, 23 July 1992, *B. Oyuntsetseg & Oyunchimeg* (UBU0031855) • Khentii province, Tsenkhermandal soum, Batkhairkhan mountain, 47°42'30"N, 109°05'34.8"E, 1459 m, 28 May 2020, *D. Munkhtulga* (UBU0031854).

#### ﻿ Astragalus teshigicus

Taxon classificationPlantaeFabalesFabaceae

D.Munkhtulga & S.Baasanmunkh sp. nov.

E2F5EB61-737B-5207-8F27-CF21822D0E1A

urn:lsid:ipni.org:names:77359974-1

Fig. 5

##### Type.

Mongolia. Bulgan province, Teshig soum, Baga Baysgalan, 49.9679, 102.5867, 1128 m, 05 June 2018, *B. Oyuntsetseg & Sh. Baasanmunkh NW11* [Holotype UBU0002138! (Fig. 6B), Isotype UBU0002139!].

##### Diagnosis.

The morphological features of *A.teshigicus* are similar to that of *A.laguroides* but differ by its raceme oblong or oblong-cylindrical (vs. ovoid), calyx rather densely covered with spreading, straight only white hairs 2 mm (vs. densely covered with asymmetrically bifurcate to basifixed, tangled, spreading white hairs 1–2 mm, at outer side of the teeth and sometimes at the nerves of the tube also with black hairs), leaves 8–18 cm (vs. 3–14 cm long), leaflets in 4–6 pairs, elliptical, 14–20 × 5–8 mm (vs. 3–5 pairs, narrowly elliptic, 8–25 × 3–6 mm) (Table 2).

**Figure 5.**
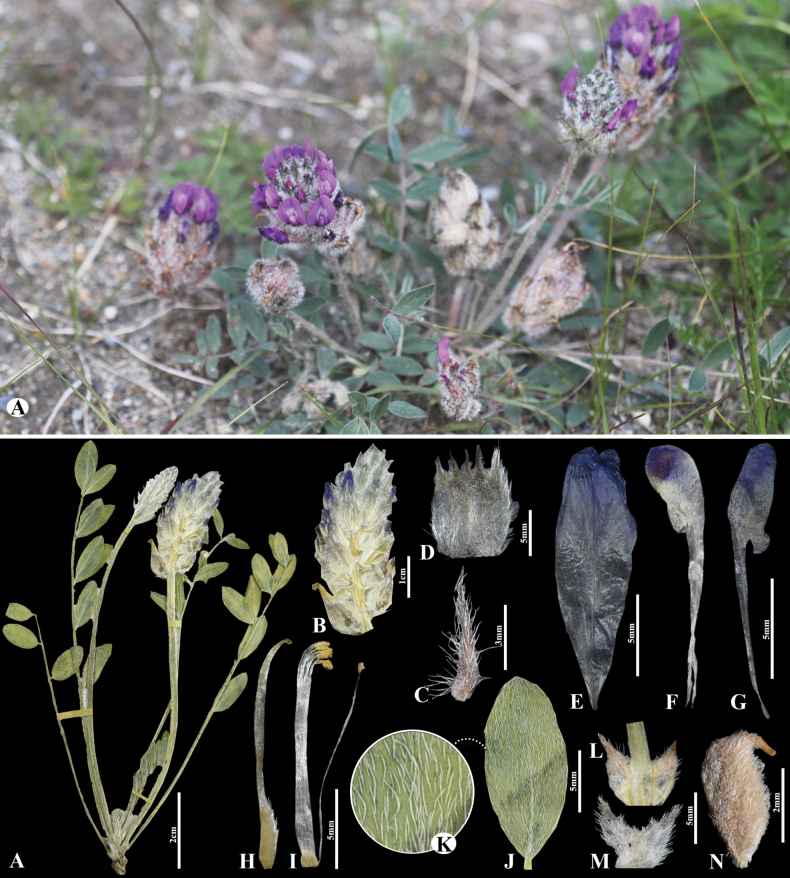
*Astragalusteshigicus* in Mongolia **A** general habit **B** raceme **C** bract **D** calyx **E** standard **F** keel **G** wings **H** pistil **I** stamens **J** leave **K** omission of medifixed hairs on the upper side of the leaf **L** stipules, inside view **M** stipules, outside view **N** pod. (Photo credits: D.Munkhtulga and B.Oyuntsetseg).

##### Description.

Plants perennial, 8−20 cm tall, acaulescent. Stems up to 1 cm, densely appressed white hairy. Stipules 4−5 mm long, broadly triangular-acuminate, densely covered with spreading or ascending hairs. Leaves 8−18 cm; petiole 3−7 cm, like the rachis covered with appressed white hairs. Leaflets in 4−6 pairs, elliptical, 14−20 × 5−8 mm, on both sides densely covered with medifixed, subappressed hairs, acute to rarely obtuse. Peduncles 6−12 cm, with medifixed, appressed hairs. Racemes oblong or oblong-cylindrical (3.5) 4−7 (8) cm long, rather densely many-flowered. Bracts scarious, 5.5−6 mm, narrowly triangular, sparsely covered with ascending only white hairs, at the margins covered with spreading white hairs up to 1.5−2 mm. Calyx 12−15 mm, at the beginning of anthesis tubular, soon ovoid-inflated, densely covered with basifixed, ± spreading, rigid white hairs 1.5−2 mm; teeth subulate, 2−3 mm. Petals purplish. Standard 20−24 mm; blade 4.5−5.5 mm wide, obovate, slightly constricted in the middle, slightly emarginate, at the base gradually narrowed. Wings 17−22 mm; blades oblong, rounded, 5−5.5 × 2−3 mm; auricle c. 1 mm; claw 8−9 mm. Keel 14−18 mm; blades 4−4.5 × 2−3 mm; claw c. 8 mm. Ovary sessile. Legumes enclosed in the calyx, oblong, 4−5.5 × 2−2.5 mm, with a hooked beak c. 1 mm, unilocular; densely covered with spreading white hairs.

**Figure 6.**
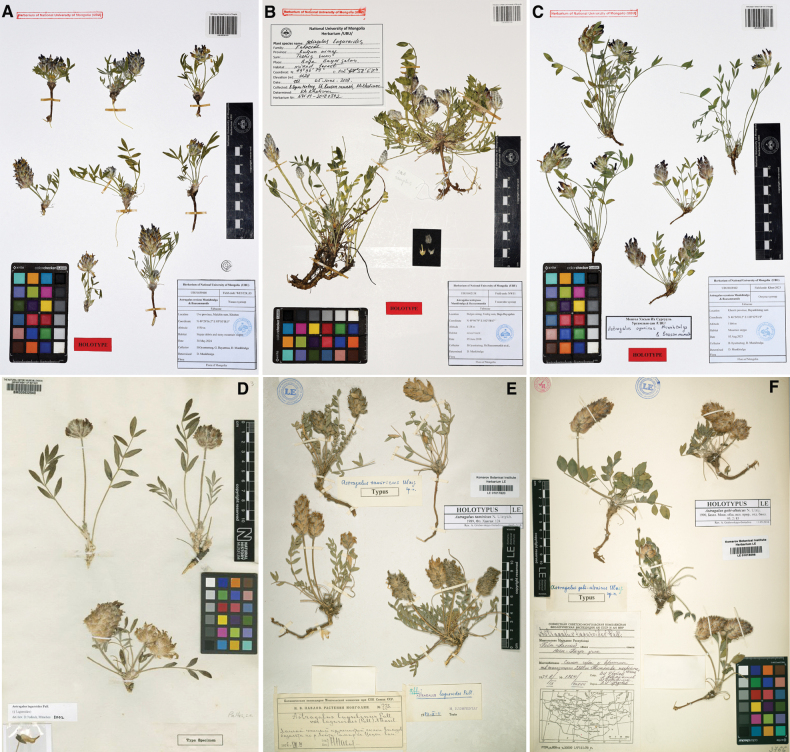
Type specimens of *Astragalus* species **A***A.uvsicus* (UBU0039100) **B***A.teshigicus* (UBU0002138) **C***A.oyunicus* (UBU0039102) **D***A.laguroides* (BM000632540) **E***A.tamiricus* (LE01017820) **F***A.gobi-altaicus* (LE01016096).

##### Distribution.

Endemic to Mongolia. This species is found only in a single location in the northern part of the country (Fig. 9).

##### Habitat.

This species grows in steppe areas including mountain slopes, rocky and stony mountains.

##### Etymology.

The species epithet refers to the location where the species was collected.

#### ﻿ Astragalus subgenus Cercidothrix Bunge, section Uvsicus

Taxon classificationPlantaeFabalesFabaceae

D.Munkhtulga, S.Baasanmunkh & H.J.Choi sect. nov.

3D1ACBF7-5DF7-5236-BCDF-5F7B5DD88BD0

urn:lsid:ipni.org:names:77359975-1

##### Type.

*A.uvsicus* D.Munkhtulga, S.Baasanmunkh & H.J.Choi, sp. nov.

##### Description.

Perennials, herbaceous, acaulescent, with bifurcate hairs. Stipules adnate, like rachis densely to rather densely hairy. Leaves with appressed hairs. Inflorescens with a well developped peduncle, densely mostly many-flowered. Bracteoles absent. Calyx tubular at beginning of anthesis, mostly soon elongating and becoming ovoid to globose-inflated. Legumes enclosed in the calyx, sessile, bilocular.

#### ﻿ Astragalus uvsicus

Taxon classificationPlantaeFabalesFabaceae

D.Munkhtulga, S.Baasanmunkh & H.J.Choi sp. nov.

8256ABF3-36C1-56A5-89AD-F791529C5A79

urn:lsid:ipni.org:names:77359976-1

Fig. 7

##### Diagnosis.

*Astragalusuvsicus* is morphologically similar to *A.beitashanensis* W. Chai & P. Yan (Zhai and Yan 2010) and *A.laguroides*, but can be distinguished by its leaflet 2–5 pairs, narrowly elliptic, acute 8–11(–14) × 3–5 mm (*A.beitashanensis*, vs. 3–5 pairs, elliptic to suborbicular, obtuse, 4–9 × 3–6 mm; vs. *A.laguroides*, 3–5 pairs, narrowly elliptic, acute 8–25 × 3–6 mm) Legumes linear, 5–6 × 2–3 mm, with a beak c. 1 mm, bilocular, loosely covered with spreading, straight hairs (*A.beitashanensis*, legumes oblong, c. 8 mm, unilocular, very densely covered with spreading, basifixed hairs; *A.laguroides*, legumes oblong, (6) 7–8 × 2–3 mm long, with a hooked beak c. 1 mm, unilocular; covered with subappressed to spreading hairs). At the section level, the Uvsicus section is differentiated by its plants covered with medifixed hairs (vs. covered with semi-appressed pilose in *Mixiotricha*), peduncle not longer than leaves, raceme many-flowered (vs. peduncle very short, raceme few-flowered in *Trachycercis* and *Mixiotricha*), legumes enclosed in the calyx (vs. calyx ruptured by legumes in *Macrotrichoides*), and bilocular (vs. unilocular in *Laguropsis*) (Table 3). The general habits of selected sections within the subgenus Cercidothrix are shown in Fig. 8.

**Table 3.** Morpholgical comparisions of five *Astragalus* sections in Mongolia.

Characters	*Uvsicus*	*Laguropsis*	*Trachycercis*	*Mixiotricha*	*Macrotrichoides*
Plants	appressed pilose	appressed pilose	appressed pilose	semiappressed pilose	appressed pilose
Raceme	many flowered	many flowered	few flowered	few flowered	few flowered
Peduncle	long	long	very short	very short	long
Calyx	vesicularly inflated	vesicularly inflated	not inflated	not inflated	slightly inflated
Pods	bilocular	bilocular and unilocular (in *A.laguroides*, *A.oyunicus*, *A.teshigicus*, *A.gobi-altaicus*)	Bilocular	bilocular	bilocular

**Figure 7.**
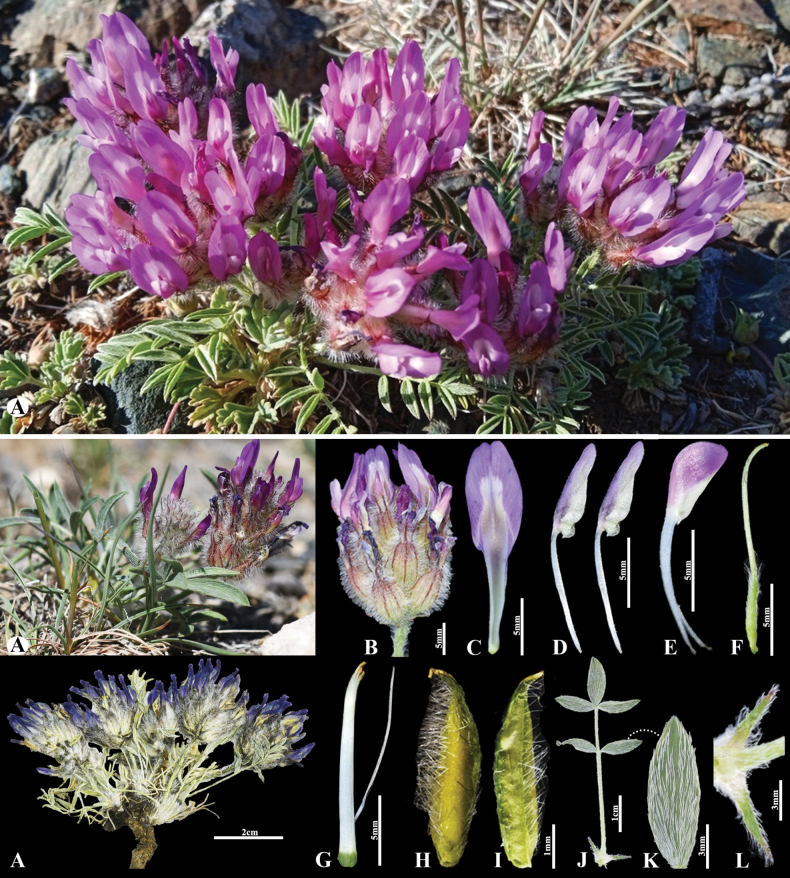
*Astragalusuvsicus* in Mongolia **A** general habits **B** raceme **C** standard **D** wings **E** keel **F** pistil **G** stamens **H** pod **I** pod valve **J** leave **K** leaflet **L** stipules. (Photo credits: D.Munkhtulga).

**Figure 8.**
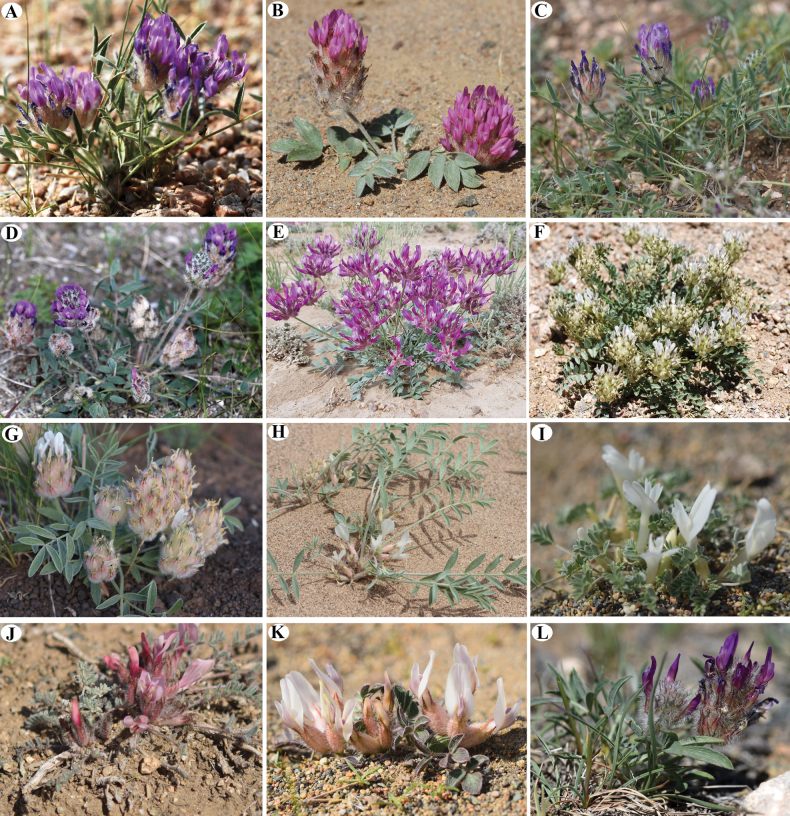
General habits of some selected *Astragalus* species in Mongolia **A***A.laguroides***B***A.gobi-altaicus***C***A.oyunicus***D***A.teshigicus***E***A.ochrias***F**. *A.dilutus***G***A.lupulinus* (**A–G** sect. Laguropsis) **H***A.junatovii* (sect. Trachycercis) **I***A.teskhemicus* (sect. Trachycercis) **J***A.testiculatus* (sect. Mixiotricha) **K***A.gubanovii* (sect. Macrotrichoides) **L***A.uvsicus* (sect. Uvsicus) (Photo credit: A, C, F, G, I, J, K, L by D. Munkhtuglga; A, B, D, E, H by B. Oyuntsetseg).

##### Type.

Mongolia. Depression of Great Lakes: • Uvs province, Malchin soum, Khuiten valley, 49°29'36.2"N, 93°10'18.5"E, 1550 m, 30 May 2024, *B. Oyuntsetseg, G. Bayarmaa, & D. Munkhtulga* [Holotype UBU0039100! (Fig. 6A)] • Uvs province, Malchin soum, Surtiin zuslan, 49°37'0.6"N, 93°3'46.6"E, 1611 m, 10 June 2021, *D. Munkhtulga* [Isotype UBU0027685!].

##### Description.

Plants perennial, 5−12 cm tall, acaulescens or nearly so, covered with medifixed hairs. Rootstock with a pluricipital root-crown. Stipules narrowly triangular-acuminate, 5−7 mm, shortly adnate to the petiole, otherwise free from each other, covered with strongly asymmetrically bifurcate, subappressed white hairs up to 2 mm. Leaves 3−8 (12) cm; petiole 2−3 cm, like the rachis covered with medifixed, appressed hairs. Leaflets in 2−5 pairs, narrowly elliptic, 8−12 × 3−5 mm, apex acute, rather densely covered with medifixed, appressed hairs c. 1 mm. Peduncles 2−6 cm, with medifixed, appressed hairs. Racemes globose or ovate 3−4 cm long. Bracts scarious, 2−3 mm, narrowly triangular, white and few black hairy. Calyx at beginning of anthesis tubular, later on ovoid-inflated, 11−14 mm, with distinct, elevated longitudinal nerves, loosely covered with subbasifixed, spreading white and black hairs 1−2 mm; teeth subulate, 2−3 mm. Petals violet. Standard 15−18 mm; blade 3−4 mm wide, obovate, slightly constricted in the middle, emarginate, at the base gradually narrowed into the rather long claw. Wings 13−15 mm; blades narrowly oblong, obtuse, 5.5−6.5 × 1.1−2 mm; auricle c. 1 mm; claw 7.5−8.5 mm. Keel 12−14 mm; blades 4−5 × 3 mm. Ovary sessile. Legumes enclosed in the calyx, linear, c. 5−6 × 2−3 mm, with a beak c. 1 mm, bilocular, loosely covered with spreading, straight white hairs c. 1 mm, smaller black hairs amount greater than the apex.

##### Distribution.

**Figure 9.**
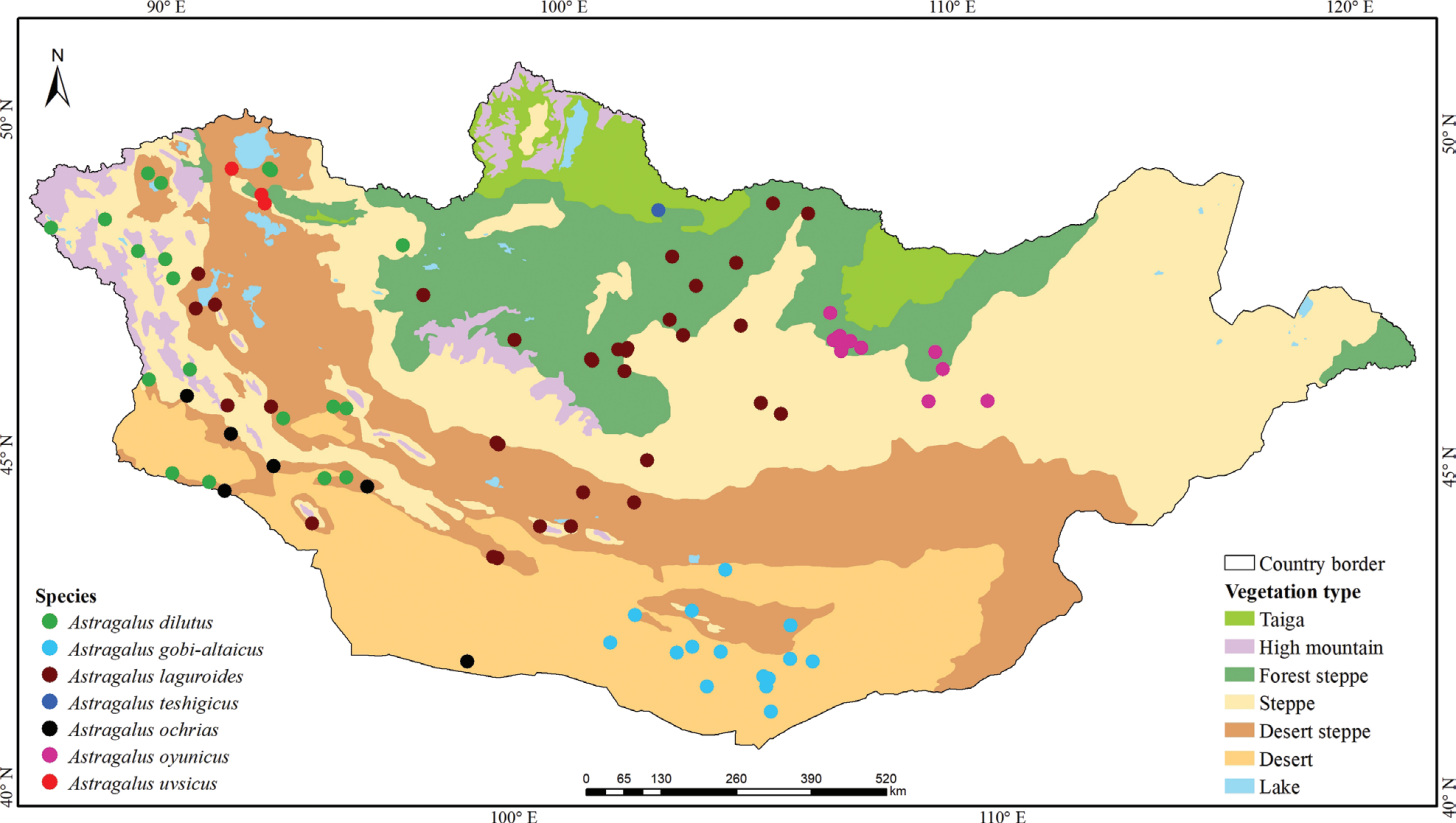
Distribution map of the studied *Astragalus* species from the sections *Laguropsis* and *Uvsicus* in Mongolia.

Endemic to Mongolia. This species is found in the Khyargas and Uvs lakes in the depression of great lakes region in Mongolia (Fig. 9).

##### Habitat.

This species grows in stony mountain slopes and near rocks, sayr slopes.

##### Etymology.

The species epithet refers to the location where the species was collected.

##### Additional specimens examined.

Mongolia. Depression of Great Lakes: • Uvs province, Tarialan soum, Turgen river, 49°56'10.9"N, 92°15'22.7"E, 900 m, 31 August 1984, *I. A. Gubanov 9338* (MW0183285).

## ﻿Conclusion

Based on morphological characteristics and molecular analysis, we described three new species, *A.oyunicus*, *A.teshigicus*, and *A.uvsicus*, from Mongolia in this study. In addition, a new section of *Uvsicus* is described based on *A.uvsicus*, supported by morphological and molecular evidence. We conclude that morphological characteristics and size are important for distinguishing between closely related *Astragalus* species. In addition, we confirmed the finding of several previous studies (Bagheri et al. 2016; Zhang and Jiang 2020; Baasanmunkh et al. 2024a) that the nrITS primer is quite well distinguished between species at the section and subgenus levels. Finally, we partially studied the species of the section Laguropsis; however, we will continue to study the remaining species in Central Asian countries.

## Supplementary Material

XML Treatment for Astragalus laguroides

XML Treatment for Astragalus gobi-altaicus

XML Treatment for Astragalus oyunicus

XML Treatment for Astragalus teshigicus

XML Treatment for Astragalus subgenus Cercidothrix Bunge, section Uvsicus

XML Treatment for Astragalus uvsicus

## ﻿Additional information

### Author ORCIDs

Dariganga Munkhtulga https://orcid.org/0009-0009-0175-203X

Shukherdorj Baasanmunkh https://orcid.org/0000-0003-4224-9376

Nudkhuu Nyamgerel https://orcid.org/0000-0002-9110-423X

Jong Ho Park https://orcid.org/0009-0003-9053-8591

Zagarjav Tsegmed https://orcid.org/0000-0002-6024-4918

Komiljon Sh. Tojibaev https://orcid.org/0000-0003-2846-5777

Hyeok Jae Choi https://orcid.org/0000-0001-6315-0071

### Data availability

All of the data that support the findings of this study are available in the main text.
